# Hypodermic Injections of Carbolic Acid in Neuralgias

**Published:** 1877-01

**Authors:** 


Hypodermic Injections of Carbolic Acid in Neuralgias.—We find the following remarks on tne above treatment in *Allgemeine Medicinische Central Zeitung,* No. 72, 1876, contributed by Dr. Merten, of Neuwedell.

In Nos. 61 and 62, 1875, of this periodical, Dr. Schulz published a few cases of neuralgia cured by the subcutaneous injection of a solution of carbolic acid. I have recently had the opportunity of treating several cases similarly, and with beneficial results; a few of the most important will now be given.

1. Miss B., aged nineteen years, slender but not chlorotic, has been treated by me during more than a year, for manifold hysterical symptoms. By degrees the left eye became affected with almost complete hemiopia, as well as strabismus and ptosis, so that the slit between the lids of that eye presented a very contracted appearance. Repeated examinations with the ophthalmoscope gave no results. Injections of strychnine in the temporal region produced temporary improvement sometimes. After some months severe neuralgia suddenly attacked the malar branch of the facial nerve on the affected side. Three-fourths of a Pravaz syringeful of a two per centum solution of carbolic acid was injected subcutaneously into the neighborhood of the nerve. This produced intense burning pain, with great redness and swelling of the integument at the point of injection.

*After the expiration of one hour, there was a complete cessation of all pain.* On the next morning I observed that the strabismus and ptosis had vanished entirely, and that no pain whatever remained; the patient also stated that the hemiopia had become much less marked. I proposed a second injection for the latter symptom, but the patient has not yet reappeared.

2. Mrs. S., aged thirty-three years, had a sudden rigor and fever, from an attack of *ovaritis sinistra.* I was consulted on the third day, and found the patient suffering with severe pain in the anterior portion of the left hip; ordered applications of ice to the ovarial region, and morphine internally. Next day the ovary was less sensitive; the pain had grown more severe in the external cutaneous nerve of the thigh, and there was almost unendurable pain in the region of the anus while defecating. That evening gave a hypoderm of one syringeful of a two per centum solution of carbolic acid in the vicinity of the left anterior superior spiness process of the ilium. No phenomena at site of injection; good sleep; no fever; pain had almost vanished the following morning. A second injection in the same region was succeeded by a lasting discontinuance of all the pains, including those in the ovary, the hip, and the anus.

Matilda K., aged twenty-four years, washed clothes in a creek for hours, while her menses were on; the same night severe pains appeared in her back, and in the posterior portion of her left leg, so that she could neither sleep nor leave her bed. Alcoholic liniments were applied for two days, with increase of pain and continuance of sleeplessness. I then saw the patient and diagnosed suppression of the menstrual flow and sciatica in the left limb. One syringeful of a $\frac{3}{100}$ solution of carbolic acid was injected subcutaneously over the gluteus maximus muscle, in a line with the point of emergence of the great sciatic nerve from the pelvis. The patient resided in the country, and was not seen again for several months, when she stated that two hours after the injection pain ceased in her leg and she went comfortably to sleep. Next day she was able to leave her bed and go about her work. At the proper time her menstrual flow reappeared.

Mr. L., aged thirty-eight years, slept all night near an open window, and had pain in the integument of the right side of his neck on the following morning. He applied the cold water bandage of Priessnitz, which increased his malady. On examination it was found that all the movements of the cervical muscles were possible without pain. A hypoderm of one Pravaz' syringeful of the $\frac{3}{100}$ solution of carbolic acid produced a cure.

Of these cases, the first will probably excite most interest, because it not only illustrates the anæsthetic action of carbolic acid, but shows that it restored the lost tonicity of the muscles involved through a stimulating action upon the nerves supplying them, a phenomenon which might well demand of us that carbolic acid injections should be given a trial in other motory disturbances.

